# Systematic review and meta-analysis of the efficacy of breast conservation therapy followed by radiotherapy in four breast cancer subtypes

**DOI:** 10.18632/oncotarget.18205

**Published:** 2017-05-24

**Authors:** Xin-Bin Pan, Rou-Jun Chen, Shi-Ting Huang, Yan-Ming Jiang, Xiao-Dong Zhu

**Affiliations:** ^1^ Department of Radiation Oncology, Cancer Hospital of Guangxi Medical University, Nanning, Guangxi 530021, P.R. China

**Keywords:** breast cancer, molecular subtypes, breast conservation therapy, radiotherapy

## Abstract

The different molecular subtypes of breast cancer are associated with distinct outcomes. We assessed the efficacy of breast conservation therapy (BCT) followed by radiotherapy for patients with different breast cancer subtypes. We searched the MEDLINE, EMBASE, and Cochrane Library databases to identify studies published prior to April 30, 2016 that assessed the efficacy of BCT followed by radiotherapy in breast cancer patients with different molecular subtypes. A meta-analysis of seven studies that included 3,798 luminal A, 770 luminal B, 344 human epidermal growth factor receptor 2 (Her-2), and 767 triple-negative breast cancer (TNBC) patients was performed. The pooled odds ratio [OR] for local relapse-free survival in luminal A compared to Her-2 patients was 0.1960 (95% confidence interval [CI]: 0.0440–0.8728, p = 0.0325) at 5 years and 0.2592 (95% CI: 0.1301–0.5167, p = 0.0001) at 10 years. The pooled OR for local-regional relapse-free survival in luminal A compared to TNBC patients was 0.1381 (95% CI: 0.0565–0.3374, p = 0.0000) at 5 years and 0.1221 (95% CI: 0.0182–0.8192, p = 0.0304) at 10 years. Thus, the rate of local-regional control is higher in luminal A patients than in Her-2 or TNBC patients.

## INTRODUCTION

Breast conservation therapy (BCT) followed by radiotherapy is the standard of care for early-stage breast cancer. Previous studies have evaluated the outcomes of early-stage breast cancer patients without distinguishing between subtypes. However, breast cancer is a heterogeneous disease characterized by a wide spectrum of clinical, pathological, and molecular features [1, 2]. Importantly, the molecular subtypes can predict therapeutic response and prognosis.

Several studies have suggested that luminal A breast cancer has the best prognosis, whereas human epidermal growth factor receptor 2 (Her-2) and triple-negative breast cancer (TNBC) have higher rates of local recurrence [3–6]. However, other studies have reported no differences in pairwise comparisons between molecular subtypes [7, 8]. Because there is a low risk of local recurrence for early-stage breast cancer (≤ 5% at 5 years) [9], it is difficult to assess the association between molecular subtypes and local control. We performed a systematic review and meta-analysis to evaluate the efficacy of BCT followed by radiotherapy for the treatment of the four different molecular subtypes of breast cancer: luminal A, luminal B, Her-2, and TNBC.

## RESULTS

### Study selection and characteristics

**Figure 1:**
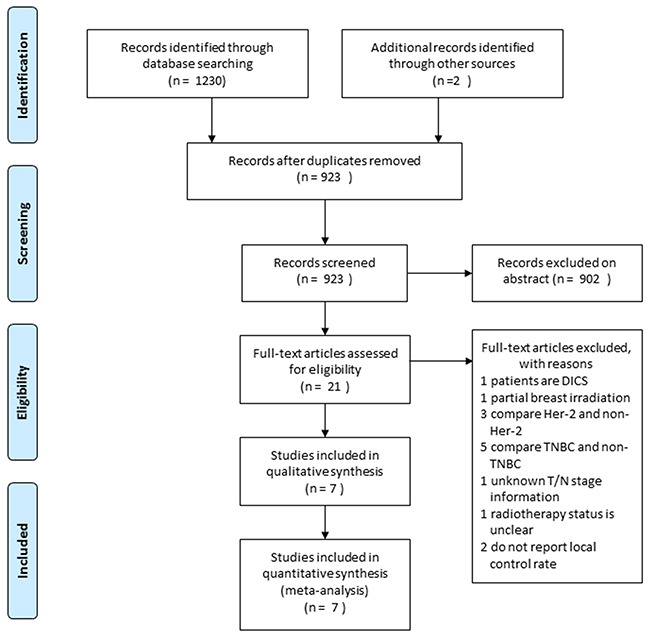
Flow chart depicting the study selection process

The study evaluation process is shown in Figure 1. A total of 1,232 titles were reviewed. Seven studies were selected for our meta-analysis [7, 8, 10–14], which included 3,798 luminal A, 770 luminal B, 344 Her-2, and 767 TNBC patients. The study characteristics are summarized in Table 1.

**Table 1:** Study characteristics

Study	Enrollment period	Population					Age (years)	Follow-up (months)	RT	CT	HT	Surgery			
		Luminal A	Luminal B	Her-2	TNBC	Total						T stage	N stage	Grade 3	Margin (+)
Arvold, 2011, USA	1997-2006	1103 (76.92%)	105 (7.32%)	55 (3.84%)	171 (11.92%)	1434	≥55 47.0%	median: 85	median: 61 Gy	660 (46.03%)	1104 (76.99%)	T3 0.8%	N3 1.0%	30.5%	2.3%
Demirci, 2012, USA	1985-2005	295 (66.59%)	75 (16.93%)	17 (3.84%)	56 (12.64%)	443	median: 56	median: 118	median: 62 Gy	66 (14.90%)	204 (46.05%)	T1-2	N0-1	nc	nc
Hattangadi-Gluth, 2011, USA	1998-2003	937 (76.61%)	98 (8.01%)	52 (4.25%)	136 (11.12%)	1223	median: 55	median: 70.4	median: 60 Gy	558 (45.63%)	942 (77.02%)	T1-2	N3 1.1%	31.6%	3.9%
Sanpaolo, 2011, Italy	2000-2008	361 (46.64%)	124 (16.02%)	134 (17.31%)	155 (20.03%)	774	median: 55	median: 59	median: 60 Gy	328 (42.38%)	186 (24.03%)	T1-2	N0-2	26.9%	9.0%
Wong, 2011, Singapore	1989-2007	247 (59.81%)	76 (18.40%)	34 (8.23%)	56 (13.56%)	413	median: 49	median: 72	median: 60 Gy	194 (46.97%)	369 (89.35%)	T3 0.4%	nc	30.7%	4.8%
Millar, 2009, Australia	1996-2003	394 (79.12%)	23 (4.62%)	13 (2.61%)	68 (13.65%)	498	median: 61	median: 84	50/61 Gy	117 (23.49%)	223 (44.78%)	T3 0.2%	N3 0.4%	29.1%	nc
Bane, 2014, Canada	1993-1996	461 (51.57%)	269 (30.09%)	39 (4.36%)	125 (13.98%)	894	≥50 73.6%	median: 144	42.5/50 Gy	110 (12.30%)	384 (42.95%)	T1-2	N0	20.0%	nc

RT: radiotherapy, CT: chemotherapy, HT: hormone therapy, nc: not clear.

### Quality assessment

The methodological quality of the included studies is shown in Table 2. An independent assessment of outcome parameters was performed in two studies [8, 13]. Outcome parameters were unclear in five studies [7, 10–12, 14]. One study did not have clearly defined outcomes [12].

**Table 2:** Methodology quality assessment

Criteria	Studies						
	Arvold	Demirci1	Hattangadi-Gluth	Sanpaolo	Wong	Millar	Bane
Clear definition of study population	√	√	√	√	√	√	√
Clear definition of outcomes and outcome assessment	√	×	√	√	√	√	√
Independent assessment of outcome parameters	?	?	√	?	?	√	?
Sufficient duration of follow-up	√	√	√	√	√	√	√
No selective loss during follow-up	√	√	√	√	√	√	√
Important confounders and prognostic factors identified	√	√	√	√	√	√	√

√: yes; ×: no; ?: not clear.

### Meta-analysis

The pooled odds ratios [ORs] for patients with the different molecular subtypes who were treated with BCT followed by radiotherapy are shown in Table 3. The analysis of study heterogeneity and publication bias are also presented in Table 3.

**Table 3:** Pooled odds ratios for BCT followed by radiotherapy on different molecular subtypes of breast cancer

Outcome	Comparison	Meta-analysis				Heterogeneity test			Model	Publication bias (p Value)	
		OR	95%CI	z Value	p Value	I^2^	q Value	p Value		Begg's test	Egger's test
5-year LFS											
	Luminal A vs. luminal B	0.5221	0.2815-0.9684	2.06	0.0392	0.00	3.39	0.4955	Fixed	0.8065	0.7773
	Luminal A vs. Her-2	0.1960	0.0440-0.8728	2.14	0.0325	79.98	19.98	0.0005	Random	0.8065	0.7624
	Luminal A vs. TNBC	0.1731	0.0674-0.4444	3.65	0.0003	73.14	14.89	0.0049	Random	0.0864	0.2480
	luminal B vs. Her-2	0.4306	0.0741-2.5006	0.94	0.3478	72.05	14.31	0.0064	Random	0.8065	0.1702
	luminal B vs. TNBC	0.3588	0.0872-1.4755	1.42	0.1554	69.43	13.09	0.0109	Random	1.0000	0.0232
	Her-2 vs. TNBC	0.9483	0.5497-1.6357	0.19	0.8486	24.92	5.33	0.2553	Fixed	0.4624	0.2496
5-year LRFS											
	Luminal A vs. luminal B	0.4670	0.0610-3.5768	0.73	0.4635	-	-	-	Fixed	-	-
	Luminal A vs. Her-2	0.1320	0.0310-0.5613	2.74	0.0061	-	-	-	Fixed	-	-
	Luminal A vs. TNBC	0.1381	0.0565-0.3374	4.34	0.0000	-	-	-	Fixed	-	-
	luminal B vs. Her-2	0.2826	0.0283-2.8247	1.08	0.2820	-	-	-	Fixed	-	-
	luminal B vs. TNBC	0.2957	0.0400-2.1857	1.19	0.2325	-	-	-	Fixed	-	-
	Her-2 vs. TNBC	1.0462	0.2586-4.2316	0.06	0.9495	-	-	-	Fixed	-	-
10-year LFS											
	Luminal A vs. luminal B	0.5860	0.3422-1.0037	1.95	0.0516	0.00	0.27	0.6048	Fixed	1.0000	-
	Luminal A vs. Her-2	0.2592	0.1301-0.5167	3.84	0.0001	0.00	0.42	0.5177	Fixed	1.0000	-
	Luminal A vs. TNBC	0.6887	0.2341-2.0261	0.68	0.4981	62.39	2.66	0.1030	Random	1.0000	-
	luminal B vs. Her-2	0.4434	0.2200-0.8938	2.27	0.0230	0.00	0.80	0.3717	Fixed	1.0000	-
	luminal B vs. TNBC	1.6545	0.7414-3.6919	1.23	0.2189	0.00	0.56	0.4555	Fixed	1.0000	-
	Her-2 vs. TNBC	2.6296	0.4828-14.3219	1.12	0.2636	57.09	2.33	0.1269	Random	1.0000	-
10-year LRFS											
	Luminal A vs. luminal B	0.7707	0.2201-2.6986	0.41	0.6838	0.00	0.53	0.4667	Fixed	1.0000	-
	Luminal A vs. Her-2	0.3354	0.0980-1.1484	1.74	0.0819	0.00	0.04	0.8512	Fixed	1.0000	-
	Luminal A vs. TNBC	0.1221	0.0182-0.8192	2.17	0.0304	86.40	7.35	0.0067	Random	1.0000	-
	luminal B vs. Her-2	0.5652	0.0899-3.5525	0.61	0.5430	-	-	-	Fixed	-	-
	luminal B vs. TNBC	0.1502	0.0062-3.6671	1.16	0.2449	76.24	4.21	0.0402	Random	1.0000	-
	Her-2 vs. TNBC	0.4448	0.0489-4.0442	0.72	0.4719	54.42	2.19	0.1386	Random	1.0000	-

The pooled OR for 5-year LFS for patients with luminal A compared to luminal B, Her-2, and TNBC was 0.5221 (95% confidence interval [95% CI]: 0.2815–0.9684, p = 0.0392), 0.1960 (95% CI: 0.0440–0.8728, p = 0.0325), and 0.1731 (95% CI: 0.0674–0.4444, p = 0.0003), respectively. The pooled OR for 10-year LFS for patients with luminal A compared to Her-2 breast cancer was 0.2592 (95% CI: 0.1301–0.5167, p = 0.0001). The pooled OR for 10-year LFS for patients with luminal B compared to Her-2 breast cancer was 0.4434 (95% CI: 0.2200–0.8938, p = 0.0230).

The pooled OR for 5-year LRFS for patients with luminal A compared to Her-2 and TNBC was 0.1320 (95% CI: 0.0310–0.5613, p = 0.0061) and 0.1381 (95% CI: 0.0565–0.3374, p = 0.0000), respectively. The pooled OR for 10-year LRFS for patients with luminal A compared to TNBC was 0.1221 (95% CI: 0.0182–0.8192, p = 0.0304). There were no differences in pairwise comparisons between the other groups.

## DISCUSSION

The risk of local recurrence risk in patients with early-stage breast cancer is primarily assessed based on clinicopathological factors. We demonstrated that patients with the luminal A subtype have a better prognosis than those with the Her-2 subtype following BCT and radiotherapy. Thus, the different molecular subtypes are associated with distinct local control rates and can predict prognosis.

Although breast cancer patients are widely classified into the luminal A, luminal B, Her-2, and TNBC molecular subtypes in clinical practice [6, 15], these subtypes are only an approximation of the underlying genotype-based subtypes. A previous study suggested that a fraction of luminal B patients were misclassified as luminal A, because only 30–50% of patients classified as luminal B by genotyping were Her-2+ [6]. However, genetic analysis is often impractical in clinical practice because of the time and expense required. Therefore, clinicians routinely make treatment decisions based on classic prognostic factors.

The overall quality of the studies in our meta-analysis was moderate-to-high. The independent assessment of outcome parameters was unclear in five studies [7, 10–12, 14], which was the main source of bias in our analysis. However, independent assessment was not necessarily needed for the included studies because local recurrence is an objective outcome that is defined by pathology and imaging.

Our meta-analysis suggests that Her-2 patients have increased odds of 5- and 10-year LFS compared to luminal A patients. Trastuzumab is the preferred treatment for Her-2+ (luminal B and Her-2) patients. It has been shown to improve overall survival in Her-2+ patients with early-stage breast cancer [16]. However, local recurrence was not assessed [17, 18]. Sanpaolo et al. [7] reported similar local control rates for Her-2 and luminal A patients treated with trastuzumab. However, some studies have reported that trastuzumab did not affect the rate of local relapse in Her-2 patients, and there was no difference in local recurrence between patients with the Her-2 and luminal A subtypes [8, 19, 20]. Only two studies included in our meta-analysis analyzed patients treated with trastuzumab (87.3% had Her-2 type breast cancer in the Sanpaolo et al. study [7] and one had Her-2 breast cancer in the Wong et al. study [14]). Trastuzumab therapy was not administered to Her-2 patients in the other five studies [8, 10–13]. The observed differences in LFS between Her-2 and luminal A patients in our meta-analysis could be explained by the treatment of Her-2 patients with adjuvant trastuzumab therapy.

A previous meta-analysis demonstrated that the 5-year local control rate of TNBC patients was similar to that of non-TNBC patients [21]. In this study, TNBC patient outcomes were not analyzed according to subtype. Our meta-analysis suggests that TNBC patients had lower LRFS compared to luminal A patients. The increased risk of local-regional recurrence in TNBC patients may be correlated with the aggressive clinicopathological features and ineffectiveness of endocrine therapy or trastuzumab. There is insufficient data regarding the relationship between luminal B, Her-2, and TNBC and the risk of local recurrence. Our meta-analysis indicated there were no differences between patients with luminal B, Her-2, and TNBC.

Most local recurrences of breast cancer occur during the first 5 years following BCT and radiotherapy [22]. Our meta-analysis suggests that the local-regional control rate is higher in patients with the luminal A compared to Her-2 and TNBC. However, it is unclear whether local recurrence reflected inadequate patient selection/inadequate local treatment or aggressive disease. Her-2 and TNBC are associated with substantially higher rates of local-regional recurrence, suggesting that these subtypes are more aggressive than the luminal A subtype. Trastuzumab therapy for Her-2 patients may reduce the rate of local-regional recurrence. There are currently no effective therapies for TNBC.

Our meta-analysis had several limitations. First, the surgical approach and treatments (e.g. chemotherapy and hormone therapy) differed between studies. Some studies included patients with a positive margin and stage T3/N3 disease. The percent of patients who received chemotherapy and hormone therapy varied between studies. Second, our sample size was limited. This is because there is a low risk of local recurrence in early-stage breast cancer patients.

In conclusion, breast cancer molecular subtypes predict outcomes and treatment response in early-stage breast cancer patients treated with BCT followed by radiotherapy. Our results should be validated in patients with Her-2+ breast cancer treated with trastuzumab.

## MATERIALS AND METHODS

### Study design

Our study was performed in accordance with the Meta-analysis of Observational Studies in Epidemiology [23] and PRISMA guidelines [24].

### Selection criteria

Studies that assessed the efficacy of BCT followed by radiotherapy in breast cancer patients were reviewed. The inclusion criteria were (1) invasive breast cancer treated with BCT followed by radiotherapy, (2) complete data on breast cancer molecular subtype, and (3) at least one outcome report (i.e. local relapse-free survival [LFS]), local-regional relapse-free survival (LRFS), or sufficient information to calculate LFS or LRFS. The exclusion criteria were (1) ductal carcinoma in situ, (2) incomplete data on molecular subtype, and (3) incomplete data on BCT or radiotherapy.

Breast cancer patients were classified into the following four groups based on estrogen receptor (ER), progesterone receptor (PR), and Her-2 status: luminal A (ER+ and/or PR+ and Her-2-), luminal B (ER+ and/or PR+, and Her-2+), Her-2 (ER- and PR-, and Her-2+), and TNBC (ER-, PR-, and Her-2-) [6, 15]. Because BCT and radiotherapy mainly provide local control, the endpoints of our meta-analysis were LFS and LRFS.

### Data sources and queries

We searched the MEDLINE, EMBASE, and Cochrane Library databases to identify relevant studies completed before April 30, 2016. Search terms included ‘breast cancer’, ‘breast conservation therapy’, ‘radiotherapy’, and ‘molecular subtype’. Article reference lists were also reviewed to identify additional studies. No language restrictions were imposed.

### Study selection and quality assessment

Study selection, data extraction, and quality assessment were performed by two independent investigators. Differences were resolved through discussion with a third investigator. Quality assessment was performed according to Hayden et al. [25]. Study quality and risk of bias were assessed based on the following criteria: (1) clear definition of study population, (2) clear definition of outcomes and outcome assessment, (3) independent assessment of outcome parameters, (4) sufficient duration of follow-up, (5) no selective loss during follow-up, and (6) important confounders and prognostic factors identified.

### Statistical analysis

Cochran Q tests and I^2^ statistics were used to assess study heterogeneity, where a *p* < 0.05 and I^2^ > 50% were indicative of significant heterogeneity. A random-effects model was selected if heterogeneity was present and a fixed-effects model was selected in the absence of significant heterogeneity. Publication bias was evaluated using a funnel plot of trial effect size vs. standard error [26, 27]. All statistical analyses were performed using STATA version 12.0 (STATA, College Station, TX, USA).

## Abbreviations

BCT: breast conservation therapy
Her-2: human epidermal growth factor receptor 2
OR: odds ratio
LFS: local relapse-free survival
LRFS: local-regional relapse-free survival
ER: estrogen receptor
PR: progesterone receptor
